# Perleche, a Disease Supposed to Be New

**Published:** 1886-09

**Authors:** 


### *PERLÈCHE; A DISEASE SUPPOSED TO BE NEW*

M. Justin Lemaistre, professor at the *Ècole de Mèdecine*, at Limoges, describes a contagious affection occuring among school children in some country districts in France, which he thinks has never before been described, although the peasants have known it for some time under the name of *perlèche.* It is of a comparatively frequent occurence in the country districts of Limousin. It appears at the angles of the mouth, as a small abrasion, which in a short time extends along the labial commissures, forming cracks, or fissures, which give rise to pain and some hæmorrhage when the mouth is opened wide. The lesion has many of the objective characters of certain mucous patches and commissural rhagades in syphilitic children. The malady is a simple one, without constitutional disturbance, and usually lasts from fifteen to thirty days.

Lemaistre has frequently examined the exudation on the patches with the microscope, and unhesitatingly pronounces the affection due to a schizomycetous parasite of the globular group of bacteria. He has successfully cultivated the microbe according to the most recent methods. In addition to the spheroidal bacteria, he has detected, in the cultivation products, numerous long chaplets, or streptocci, having the power of very rapid multiplication. To the latter he has given the name *Streptococcus plicatilis.* Following up this line of investigation, he examined the drinking water used at the different schools in the district. In one of the schools the disease was first noticed in a child, who lived at some distance. The water supply at this child's home came from a fountain, which was found to contain sterptococci in abundance. Under cultivation, these microbes behaved exactly like those from the patches on the affected children. The spread of the disease among the children, at the school, was now easily explained. The pupil, who lived at a distance, had deposited on the common drinking utensil some of the germs swarming in his labial folds, which he had obtained from the water he drank at home, and thus infected the rest of the children, who had to drink from the same utensil. Out of 5,500 children, attending the thirty-two primary schools of Limoges, examined by author, 312 (1 in 17) were affected with *perlèche.* The treatment, like the disease, is simple; merely touching the patches with sulphate of copper, or alum, is quite sufficient to insure a cure.

The foregoing is particularly interesting to those who pay any attention to school-hygiene, and forcibly illustrates the evil of allowing a number of children to make use of the same cup or mug. Any question as to whether the author's assertions that the *Streptococcus plicatilis* is the cause of the disease, and that the affection has not before been known to the profession, will stand criticism, and does not detract from the weight of his argument—that the school-hygiene is sadly neglected in France—may we not add, in America, also? As to the first assertion, we fully concur in a suggestion made in a recent number of the *Progrês Mêdical*, that the author could have settled the point by inoculating one of the unaffected children with the cultivated microbes, and, considering the mildness of the affection, he would have been fully justified in doing so. Bearing in mind the numerous bacteria of various forms, that find a lodgment in the healthy mucous membrane of the mouth, we must hesitate for imputing special pathogenetic properties to any individual form.

The stomatitis described by Bergeron, 1855, as occuring in an epidemic from among soldiers, as the result of over-crowding and contagion, seems to have been much severer than *perléche.* It was attended with some febrile re-action, and local changes first appeared on the gums, from which they extended to the labial commissures. We know the predilection herpes has for the commissures, and how in a short time the little vesicles burst, run together, and form painful fissures in these situations; and for the contagiousness of some forms of herpes there is no lack of authority. Vogel does not even consider it essential for its propagation that any of the affected persons should have suffered from herpes, orginally, thus implying that it may arise from mere dirtiness. While not asserting that *perléche* and herpes abials at the commissures are identical, we think their similarity closel enough to call for further investigation before accepting M. Lemaistre's proposed addition to nosology.—*New York Medical Journal.*

